# Comment on Lee et al. Accuracy of New Deep Learning Model-Based Segmentation and Key-Point Multi-Detection Method for Ultrasonographic Developmental Dysplasia of the Hip (DDH) Screening. *Diagnostics* 2021, *11*, 1174

**DOI:** 10.3390/diagnostics12071738

**Published:** 2022-07-18

**Authors:** Sadettin Çiftci, Bahattin Kerem Aydin

**Affiliations:** 1 Department of Orthopaedics and Traumatology, Orthopaedic Surgeon, Selcuk University Faculty of Medicine, Konya 42065, Turkey; bkaydin@selcuk.edu.tr; 2 Department of Orthopaedics and Traumatology, Alaeddin Keykubat Campus, Selçuk University Faculty of Medicine, Konya 42100, Turkey

We have read the article titled “Accuracy of New Deep Learning Model-Based Segmentation and Key-Point Multi-Detection Method for Ultrasonographic Developmental Dysplasia of the Hip (DDH) Screening” by Lee et al. with great interest [1]. This paper focused on the usability of an artificial intelligence (AI)–computer aided detection (CAD) system for screening and diagnosis of DDH, for which the authors used Graf’s method to evaluate the ultrasonographic results. Although there are no errors in the statistical or AI methods used in the study, we think that there are some drawbacks in the use of the sonographic method.

Firstly, in the sonographic method used, four key points and the parallel iliac wing were used to label an image as detectable, whereas according to Graf’s method, the ultrasonographic image should include eight anatomical key points: the chondro-osseous junction, the femoral head, the synovial fold, the joint capsule, the acetabular labrum, the hyaline cartilaginous preformed acetabular roof, the bony part of acetabular roof and the bony rim (turning point from concavity to convexity). Each of these eight points is necessary to label an image detectable according to Graf’s method [2].

Secondly, there is an issue in the measurement of the Beta angle. The Beta angle was measured by the authors between the base line (iliac wing) and a second line, which runs from the end point of the base line to the labrum. According to Graf’s method, the Beta angle should be measured between the base line and an inclination line, drawn from the bony rim to the labrum [2,3]. The Beta angle measured by the authors has been described by Graf as the most common mistake in measuring the Beta angle; a missing inclination line indicates that the examiner has not correctly identified the bony rim or the labrum, which are mandatory for the image to be considered detectable.

Consequently, we think that the sonographic images and Beta angle measurements in this article are inappropriate for the evaluation of reliability of the AI-CAD system in DDH screening and diagnosis.
